# Sex-dependent metabolic and behavioural alterations in a rat model of forced exertion-induced myopathy

**DOI:** 10.1186/s12917-025-04650-x

**Published:** 2025-03-22

**Authors:** Crystal Lubbe, Brian H. Harvey, Francois P. Viljoen, Leith Meyer, De Wet Wolmarans

**Affiliations:** 1 Division of Pharmacology, Centre of Excellence for Pharmaceutical Sciences, North-West University, Potchefstroom, South Africa; 2 Centre for Veterinary Wildlife Research and Department of Paraclinical Sciences, University of Pretoria, Pretoria, South Africa; 3 MRC Research Unit on Risk and Resilience in Mental Disorders, University of Cape Town, Cape Town, South Africa; 4 Institute for Mental and Physical Health and Clinical Translation, School of Medicine, Deakin University and Barwon Health, Geelong, Australia

**Keywords:** Contextual reminder, Time-dependence, Capture myopathy, Rhabdomyolysis, Myoglobin, Anxiety

## Abstract

**Background:**

Mass boma capture (MBC) of ungulates may trigger a metabolic condition known as capture myopathy (CM), resulting in myoglobinuria and hyperthermia (rhabdomyolysis). Its pathobiology is poorly understood, especially the role of contextual reminders; a preclinical model system could thus be useful. Sixty (60) adult Sprague Dawley rats (30 rats per sex), divided into three experimental series (*n* = 12—24), were exposed to MBC-like exertion, viz., forced treadmill running (FTR) at 75% of VO_2MAX_ (30 m/min) with and without aversive noise (context) until physical exhaustion. Rectal and surface temperatures were measured before and after reaching exhaustion. Urine myoglobin, plasma lactate dehydrogenase (LDH), lactate, and creatine kinase (CK) were measured immediately and 15 days after MBC. Anxiety was assessed in the light-dark and social interaction tests.

**Results:**

Male and female MBC rats presented with significant hyperthermia, with females showing significantly increased urine myoglobin immediately after MBC, although this was not sustained until day 15 post MBC. LDH was significantly elevated in female rats at baseline but not day 15 post-MBC. Contextual re-exposure prior to testing on day 15 resulted in significant sex-dependent differences in myoglobin and CK concentrations, with female rats being significantly more affected. Only female rats trended towards increased anxiety-like behaviour immediately post-MBC exposure, which was not sustained until day 15 post MBC.

**Conclusions:**

This work builds on previous research using a rodent model of capture myopathy (CM) that confirmed the running protocol to effectively elicite the necessary muscular response. The MBC protocol emphasizes hyperthermia and increased urine myoglobin, sensitivity to contextual reminder (noise), and a trend towards anxiety, particularly in females, highlighting sex-specific physiological responses. By incorporating behavioural and biochemical assessments, acute versus delayed response and environmental triggers, the study enhances model validity and deepens insights into CM-related responses.

## Background

Large numbers of wildlife are captured and relocated annually [1]. Mass boma capture (MBC) is the most popular method for the capture of ungulates as it enables many animals to be moved over a short period of time [2]. Briefly, nets are used to construct a boma in the shape of a large funnel into which animals are driven, usually by helicopter and assisted by ground vehicles whereafter the boma opening is closed. Animals are then chased in smaller groups towards the neck of the funnel through a solidsided crush and up a loading ramp into a suitable game transport vehicle. Here, they are confined to small dark spaces, usually in the presence of other animals, but separated from their familial conspecifics [3]. All animals within the vicinity of the capturing procedure, including those already captured, are continuously exposed to human presence and audible stressors. The helicopter chase can continue for a long time, subjecting animals to extreme muscular activity and related physiological stress. Transit duration may vary from hours to days. At their destination, animals often remain highlystrung, with the new location likely acting as contextual trigger for heightened fight-or-flight activation in released animals [4, 5]. To what extent contextual re-exposure may contribute to delayed post-MBC mortality [6], remains to be established.

The pathophysiological outcomes of MBC, termed capture myopathy (CM), are multifactorial with physiological and neurocognitive sequalae [7]. First, CM can lead to significant morbidity and mortality in captured wildlife, notably ungulates. The condition is characterised by metabolic (primarily lactic) acidosis, muscle necrosis, and myoglobinuria, as well as increased plasma creatine kinase (CK), lactate dehydrogenase (LDH), and potassium concentrations [7]. These disturbances may be of either an acute or delayed nature [6], depending on the intensity and duration of the chase as well as the species involved. Myopathology is mostly related to rhabdomyolysis, a core feature of CM [7]. Furthermore, hyperthermia, hypoxia, and metabolic exhaustion also play a role in the rhabdomyolytic process [4, 8, 9]. Collectively, irreversible damage to renal and cardiac tissue ensues leading to organ failure and death. Finally, central nervous system (CNS) dysfunction [9] results in behavioural stress responses that may be short-lived or persist for weeks to months after the capture event [5, 6].

The neurocognitive stress reaction in captured ungulates involves an exacerbated state of arousal, hypervigilance, and anxiety-like fight-or-flight responses [10–12]. Since animals are unable to escape the perceived danger of MBC, uninhibited arousal and a persistent state of anxiety that is associated with excessive sympathetic nervous system activation may further contribute to the presentation of CM [13]. That said, not all animals subjected to MBC eventually progress to CM related pathology and death (4 – 10% mortality) [14]. Therefore, as is true for most stress-induced conditions in mammals [15], variances in individual susceptibility to the consequences of stress need careful consideration.

Due to a lack in understanding of the condition, the management and prevention of MBC-related complications is a significant problem for wildlife veterinarians. Novel exploratory initiatives are hampered by difficulties when studying large and often dangerous wild animals as research subjects. In this regard, a valid and translationally relevant surrogate model system could be a valuable resource for acquiring new knowledge and bolstering novel drug-intervention initiatives. Rodents are ideally suited as model subjects because of their behavioural and biological overlap with other mammals, ease of use, and high reproduction rate. As such, they represent the lowest order mammal to model CM. However, due to its complexity, any surrogate rodent model of CM would require comprehensive validation to closely reproduce the physiology and biology of the condition.

While rodent models are widely used as translational models for anxiety and stress-related research for human psychiatric disorders [16, 17], there is yet no published pre-clinical rodent model of MBC-related CM with associated anxiety and neurocognitive perturbations. For example, existing approaches to study myopathy are limited to models that rely on chemically induced rhabdomyolysis to investigate primarily the renal complications associated with muscle breakdown [18–20]. These approaches are somewhat ineffective to elaborate our understanding of CM in wildlife subjected to forced exertion.

We have recently presented provisional validation data of a rat model that emulates CM [21]. The core focus of the current investigation was to elaborate further on this model by characterising and validating its resemblance of the major pathophysiological and neurocognitive constructs of the condition as observed in wildlife. We proposed that rats subjected to MBC-like laboratory conditions would present with acute and delayed metabolic perturbations related to myopathy and skeletal muscle metabolism, i.e., rectal hyperthermia, increased urine myoglobin, plasma LDH, lactate, and CK [4, 6]. On a behavioural level, we expected increased anxiety and altered social interaction, both of which are intrinsic to MBC. Since the condition presents either as an acute or delayed manifestation in ungulates, we also explored the longitudinal biobehavioural changes in MBC-like exposed vs. non-exposed rats 24 h and 14 days after the MBC-like protocol, in the presence and absence of a contextual reminder.

## Methods

### Study layout

**Fig. 1:**
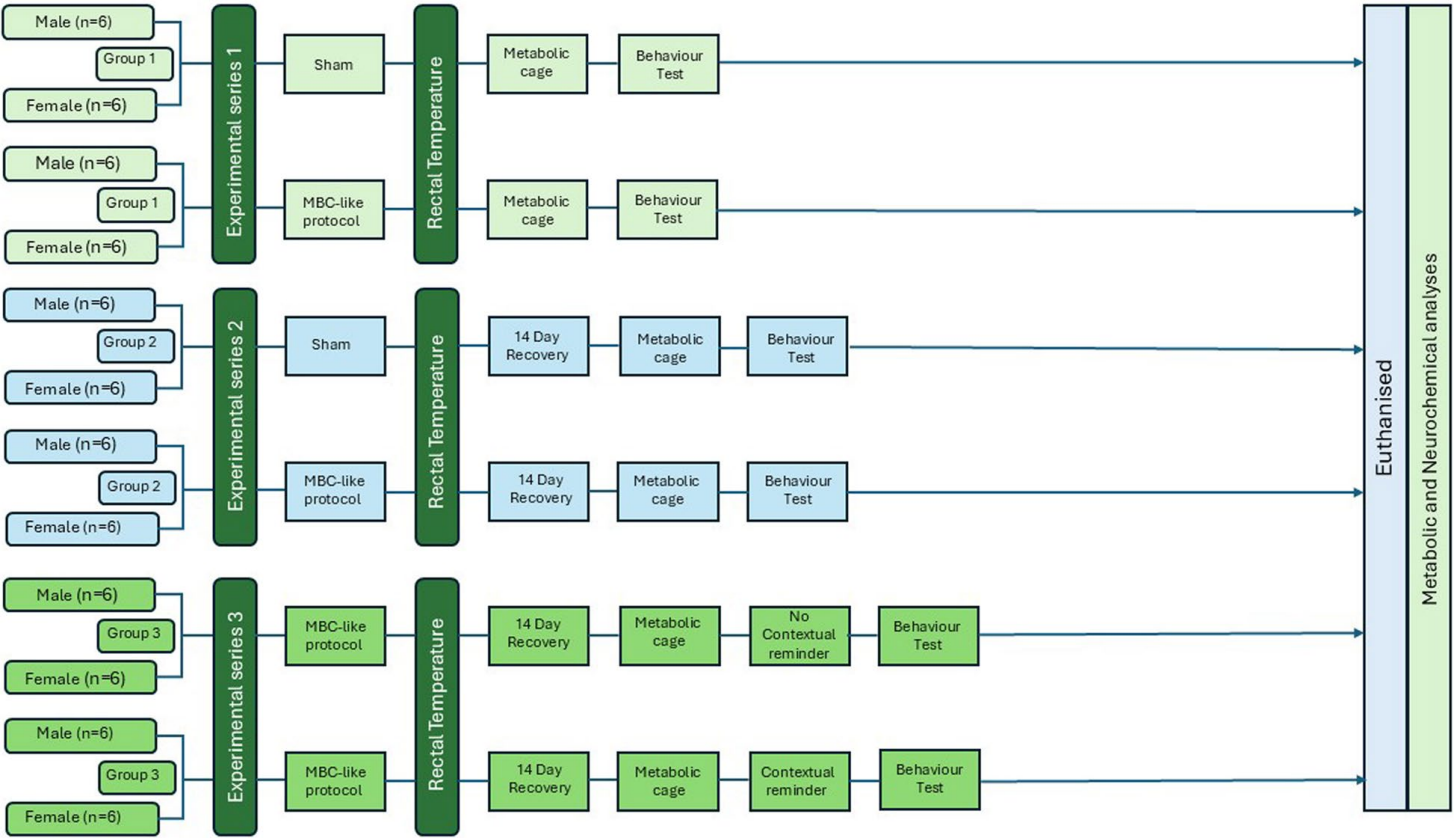
Study layout. Refer to the text for description. MBC: mass boma capture

Animal numbers were based on our first validation study (Lubbe et al. 2021) where we established an integral and quantifiable biological response in Sprague–Dawley (SD) rats following exercise, to establish an experiential basis for the unique series of stressors applied in this model. For this purpose, 12 rats per group were deemed sufficient. The study consisted of three experimental series (Fig. 1; *n* = 12—24 rats per series; see below) each designed to address the following objectives:

- Series 1: Measuring the acute behavioural, metabolic, and neurochemical responses of rats to the MBC-like protocol (immediately);
- Series 2: Measuring the delayed behavioural, metabolic, and neurochemical responses of rats to the MBC-like protocol (15 days post-MBC-like exposure); and
- Series 3: Determining the influence of an MBC-related contextual reminder (environmental trigger), i.e., aversive noise, on the delayed behavioural, metabolic, and neurochemical outcomes of MBClike-exposed rats (15 days post-MBC-like exposure).

In Series 1, rats (*n* = 24; 12 rats per group; equally distributed between sexes) were subjected to either a sham or MBC-like protocol. Immediately after sham- and MBCexposure, rectal temperatures were recorded, whereafter rats were transferred to metabolic cages for 1 h and their urine sampled. Rats were then assessed for light-dark preference and social behaviour and immediately euthanised. Trunk blood was collected and stored at minus 80 °C until further analysis.

In Series 2, rats (*n* = 24; 12 rats per group; equally distributed between sexes) were subjected to either a sham or MBC-like protocol. They were then returned to their home cages and left for 14 days to recover. On day 15 post-exposure, rats were transferred to metabolic cages for 1 h, and their urine sampled. Rats were then assessed for light–dark preference and social behaviour *without* being re-exposed to the aversive noise paired with prior MBC-like exposure and immediately euthanised. Trunk blood was collected and stored at minus 80 °C until further analysis.

In Series 3, we determined whether delayed re-exposure to the MBC-paired aversive noise was necessary to provoke or bolster the delayed outcomes of MBC-like exposure. For this purpose, an additional 12 rats (equally distributed between sexes) were exposed to the MBC-like protocol and left to recover for 14 days. On day 15, rats were exposed to MBC-likepaired with aversive noise for 20 min, after which the same procedures as described for animals in series 2, were followed. Measurements from these rats were compared with the MBC-like-exposed rats of series 2.

#### Animals

Sixty (60) adult (8–10 weeks old) SD rats (30 rats per sex) weighing approximately 150 g ± 10 g at the onset of experimentation, were sourced from twelve different breeding pairs that were initially bred, housed, and maintained at the vivarium of North-West University, South Africa (South African Veterinary Council reg. no. FR15/13458). Rats were randomly group-housed (three same-sex animals per cage randomized across litters) without litter or cage bias, i.e., each group was constituted from rats selected from different breeding pairs and rearing cages. They were housed in individually ventilated polypropylene cages (230 × 380 × 380 mm; Tecniplast®, Varese, Italy) prepared with corncob bedding. The researcher could not be entirely blinded since rats were group-exposed to the different interventions. Cages were maintained at a temperature of 22 ± 1 ºC and a humidity of 50 ± 10%. Lights were operated on a reversed 12-h light/dark cycle (lights off at 06:00 and on at 18:00) to accommodate for the experimental sequence. All experiments were conducted in the dark cycle, i.e., between 06:00 and 18:00 every day. Rats had free access to food and water throughout the investigation. The study framework, ethical considerations, statistical analyses, and final report compilation were executed inline with the ARRIVE guidelines (Kilkenny et al., 2010). All animals were maintained, and procedures performed in accordance with the approved prescriptions of the AnimCare Research Ethics Committee of North-West University (NHREC reg. no. AREC-130913–015; ethics approval number: NWU-00576-19A5).

### MBC-like protocol

#### Forced treadmill running (FTR)

The treadmill used in this investigation had six lanes on a single treadmill belt (table dimensions: 510 mm × 960 mm) which terminated on electrical grid. Animals that failed to maintain treadmill pace were exposed to electrical tail shock (1 mA, 3 Hz) [22, 23]. The running lanes, constructed of black Plexiglass®, were enclosed so that animals could not exit the apparatus during running. The speed of the treadmill could be adjusted by increments of 0.1 m/min within a range of 2.0 and 70 m/min [23].

**Fig. 2:**
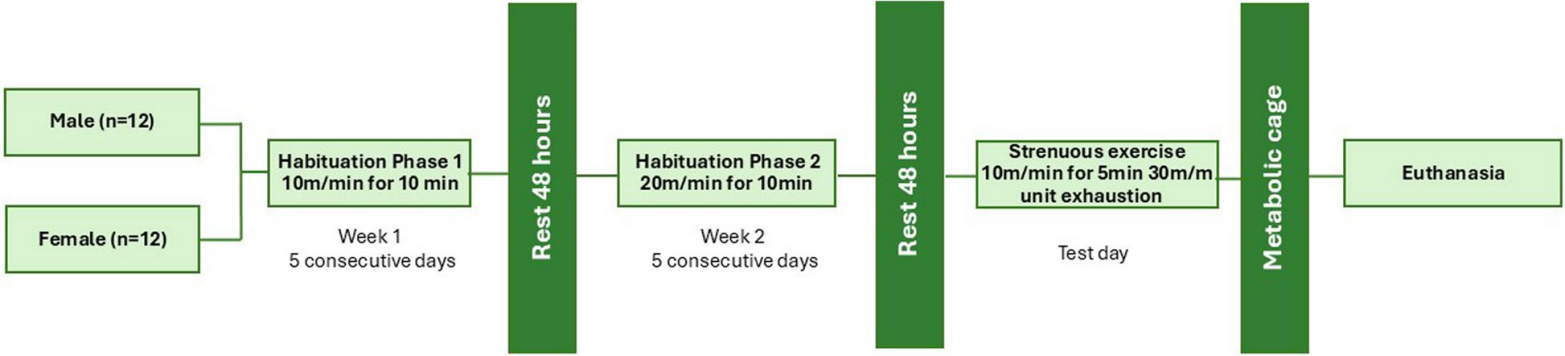
Timeline detailing the course of events of each phase of the exercise protocol followed during each week of the investigation

Prior to the onset of MBC-like exposure, all MBC-like-exposed animals were acclimatised (Fig. 2; see below) to the treadmill according to the methodology explained earlier [21]. Sham-exposed animals were merely placed on the treadmill apparatus for 10 min, without running. Briefly, rats were introduced to the treadmill for five consecutive days per week for two weeks. In week one, rats walked at a speed of 10 m/min for 10 min. In week two, the pace was increased to a speed of 20 m/min for 10 min, i.e., at 25% and 50% of the theoretical VO_2MAX_ for SD rats, respectively [24].

Two days (48 h) after the final acclimatisation session, rats were exposed to FTR. After being placed in the treadmill lanes, rats were left on the stationary belt for 1 min. The treadmill speed was set at 10 m/min for 5 min and then at 30 m/min (75% of the SD VO_2MAX_) for at least 10 min or until specific endpoints were reached. These were:

i) physical exhaustion was reached, defined as failure to remain at pace with the treadmill for the duration of the running period as indicated by four separate contacts with the electrical grid within 60 sec, or

ii) when animals chose to remain stationary, despite being exposed to electrical shock.

No rats were excluded from the study, ensuring that all data reflect the full range of physiological and behavioural responses to the MBC-like protocol.

#### White noise exposure

To mimic the sensory environment experienced by wildlife during MBC, rats were also exposed to an aversive, white noise (an anxiogenic stressor on its own; [25]), while undergoing the FTR procedure. Briefly, the white noise (played at a sound pressure of 100 dB; [24]) was produced by a single loudspeaker (15 W), driven by a white-noise generator at frequencies ranging between 80 and 9000 Hz) and monitored by a digital sound level meter (service provided by D-Media, NWU). The white noise stimulus consisted of a combination of frequencies within the mentioned range that were electronically generated and played during the experimental procedure. During Series 1 and 2, rodents were exposed to a continuous presentation of white noise during the entire MBC procedure, from the beginning of the forced exercise protocol, until after removal from confinement (see below). It was also used as a brief contextual trigger in Series 3.

#### Social confinement

To mimic the social environment experienced by wildlife during confinement and transport, rats were also socially confined post-FTR while exposed to the white noise as described above. Rats were confined in groups of three same-sex animals in small, black-walled compartments [25 cm (w) × 25 cm (d) × 25 cm (h)] for 10 min, before being returned to their home cages.

Importantly, allocation of cage mates to the running lanes during FTR and post-FTR social confinement was done in such a manner that animals were randomised across home cages so that no three familiar rats were confined together, and no two familiar rats were exposed to another in the SIT.

### Temperature measurement

Rectal temperatures of rats in Series 1 were recorded immediately after sham or MBC-like protocol to test for hyperthermia. Rectal temperatures were measured by gently inserting a lubricated (KY Jelly®, Johnson & Johnson®, Cape Town, South Africa) digital thermistor probe (Thermo-Hygro® thermometer, BAMR®, Cape Town, South Africa) to a depth of 2 cm intra-rectally until a stable reading was obtained for up to 20 s. For this procedure, rats were gently restrained at the base of the tail before the thermistor probe was inserted.

### Urine sampling and myoglobin analysis

Depending on the experimental series, urine sampling was either conducted immediately after rectal temperature measurement (Series 1) or on day 15 post-MBC-like exposure (series 2 & 3). To confirm myoglobinuria and quantify urine myoglobin content, urine samples were collected over 1 h in metabolic cages after which rats were returned to their home cages and immediately moved to the behavioural testing rooms. Food and water were provided *ad lib* during this time. Samples were immediately analysed by means of photometric detection (Indiko Plus® analyser; Thermo-Fisher Scientific®, Waltham, Massachusetts, USA). The DiaSys® Diagnostic Systems, Myoglobin FS diagnostic reagent for the quantitative in vitro determination of myoglobin was used according to the manufacturer instructions.

### Behavioural assessment

Since individual anxiety-like behaviour can vary among animals, anxiety was assessed in the light–dark test and social interaction test to increase the veracity of the findings. Depending on the experimental series, behavioural tests were conducted on the same day as sham- or MBC-like exposure (Series 1), or 15 days post-exposure (Series 2 and 3). Following urine collection, rats were left in their home cages for 10 min in the behavioural testing room (to allow for urine sample preparation and analysis).

#### Light dark test (LDT)

The LDT is based on the instinctive nature of rodents to avoid exposure to brightly lit spaces and is commonly used to assess anxiety-like behaviour [26]. However, in this work, we aimed to establish if rats would change their naturalistic preference for dark spaces after being socially confined in a small dark enclosure during the MBC-like protocol. Since the LDT arena is also a novel, potentially anxiogenic environment during a single exposure, rats could arguably enter the dark compartment due to fear of cage novelty (neophobia). To account for this possibility, and since the present work attempted to characterise post-stressor changes in normal behaviour, rats were habituated for 10 min per day over the three days preceding the day of testing. During this time, rats were allowed to freely explore both compartments of the arena.

The test setup applied in this investigation was adapted from [27]. The apparatus consisted of a dark compartment with black walls covered by a lid [15 cm (w) × 50 cm (l) × 20 cm (h)] and a large brightly illuminated compartment [30 cm (w) × 50 cm (l) × 50 cm (h)]. A small opening (8 cm × 8 cm) in the wall of the light/dark separator allowed rats to freely move between compartments. The light compartment was illuminated by bright white light (1000 lx; [26]). At the beginning of the session, rats were released in the centre of the light compartment, facing away from the opening, and allowed to explore the arena for 10 min. Time spent in the light compartment (sec) was scored and quantified with Ethovision XT® 14 software (Noldus Information Technologies®, Wageningen, The Netherlands). Arenas were cleaned with 90% ethanol and rinsed with normal water following the completion of each testing session.

#### Social interaction test (SIT)

Considering the rapid changes in social contexts that transpire during an MBC event, one of the most pressing issues encountered with wildlife translocation is anxiety due to maladaptation of translocated animals in new social structures [10, 28]. Thus, the SIT was deemed to be a potentially relevant anxiety test for the current investigation [29, 30]. Rats were also habituated to these arenas for 10 min per day over three consecutive days immediately preceding the testing day. Two unfamiliar, same-sex rats were simultaneously subjected to the SIT immediately after the LDT in a black open field arena with nontransparent walls (100 cm (w) × 100 cm (l) × 50 cm (h); [31]) and videotaped from above. Rats were left to socialise for 10 min and the net weighted movement with respect to approaching and avoidance behaviour quantified with Ethovision XT® 14 software. Net weighted movement is an objective measure of the intensity of approach and avoidance behaviour [32], allowing the average movement of subjects toward (more positive values) or away (more negative values) from each other, to be quantified over the course of a testing session. The SIT was conducted under dim red light (40 lx) [30, 33]. Arenas were cleaned with 90% ethanol and rinsed with normal water following the completion of each testing session.

### Euthanasia and plasma collection

After completion of the SIT, rats were immediately euthanised by decapitation and trunk blood collected in 4 mL EDTA-containing vacutainers (BD Vacutainer®). Samples were centrifuged at 4000 g at 4 °C for 10 min and stored at −80 °C until biochemical analysis.

### Metabolic assessment

To assess the effects of MBC-like exposure on skeletal muscle metabolism, lactate, LDH, and CK levels were determined in plasma (Indiko Plus® benchtop analyzer; Thermo-Fisher Scientific®, Waltham, Massachusetts, USA). The DiaSys® Diagnostic Systems Lactate FS, LDH 21 FS, and CK-NAC FS diagnostic reagents were used according to the manufacturer instructions.

### Statistical analysis

All statistical analyses were performed with GraphPad® Prism® (version 9; GraphPad® Software, San Diego, California). To analyse the post-rectal body temperatures of male and female SD rats, a two-way repeated measures analysis of variance (2-way RM ANOVA) was applied. Temperature was set as dependent variable, while sex and exposure were set as independent variables. To analyse the effect of MBC-like exposure on acute and delayed urine myoglobin, plasma lactate, LDH, and CK concentrations, as well as on LDT and SIT outcomes, an ordinary three-way ANOVA (3-way ANOVA) was run. Here, the respective measured parameters were set as dependent variables, while sex, time, and exposure were set as independent variables. To analyse the same parameters as above, albeit at the delayed timepoint only and in the absence or presence of a contextual trigger, an ordinary 2-way ANOVA was applied. Again, the measured outcome was set as dependent variable, while sex and contextual exposure were set as independent variables. All ANOVA analyses were followed by Bonferroni multiple comparison tests to determine the significance of pairwise differences. Statistical significance was set at *p* < 0.05 for all analyses. Where applicable, pairwise comparisons were informed with Cohen’s *d* effect size.

## Results

### Effect of MBC-like exposure on rectal temperature

**Fig. 3:**
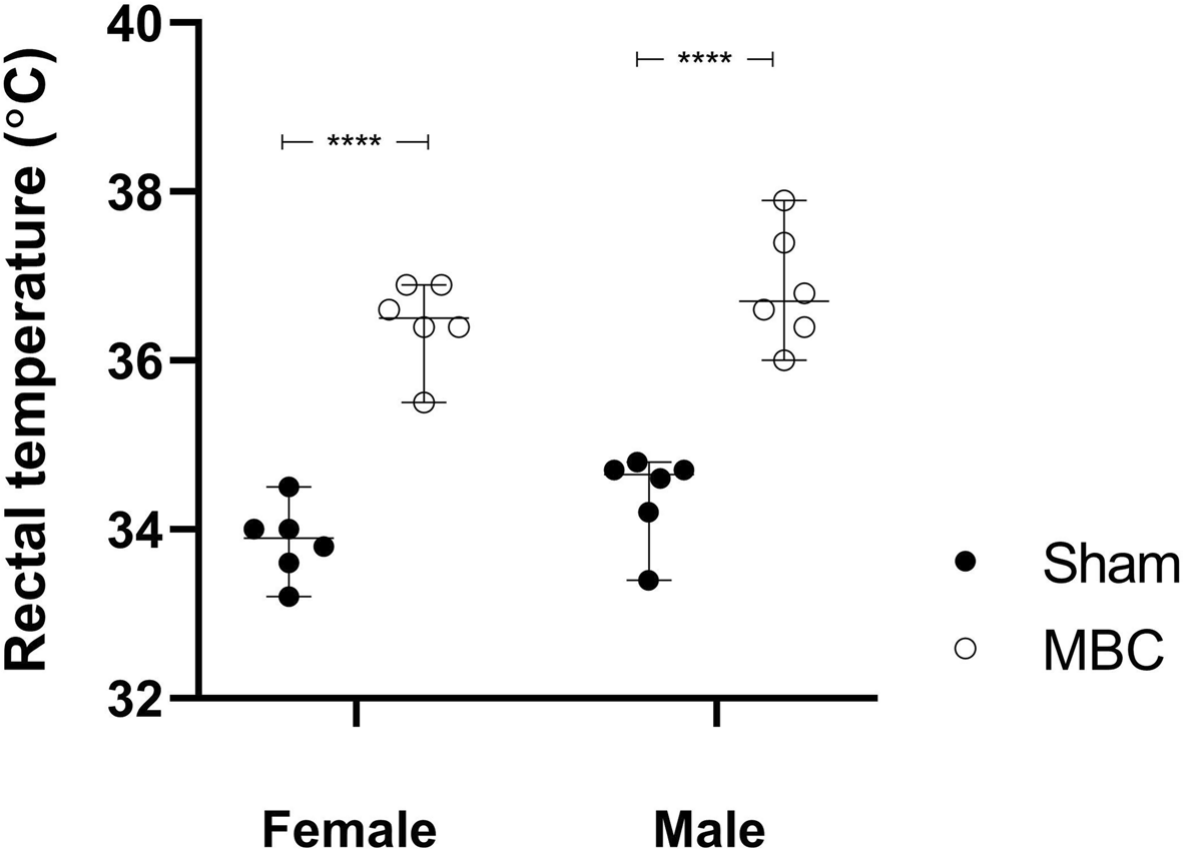
Rectal body temperatures (°C) of female and male rats of sham (black dots) and MBC-like exposed rats (open circles); ****p < 0.0001. The error bars describe the 95% confidence interval

Two-way ANOVA failed to reveal a significant exposure-sex interaction [*F*(1, 20) = 0.11, *p* = 0.74, Fig. 3]. However, both exposure [*F*(1, 20) = 125.3, *p* < 0.0001] and sex [*F*(1, 20) = 4.44, *p* = 0.05] significantly impacted the result. Subsequent pairwise comparisons revealed a significant increase in the post-exposure rectal temperatures of female (36.5 ± 0.5 vs. 33.9 ± 0.4 °C, *p* < 0.0001, *d* = 5.4, *d*CI[2.8; 8.0]) and male (36.8 ± 0.7 vs. 34.4 ± 0.5 °C, *p* < 0.0001, *d* = 4.0, *d*CI[1.9; 6.0]) rats.

### Effect of MBC-like exposure on acute and delayed urine myoglobin and skeletal muscle metabolites

**Fig. 4:**
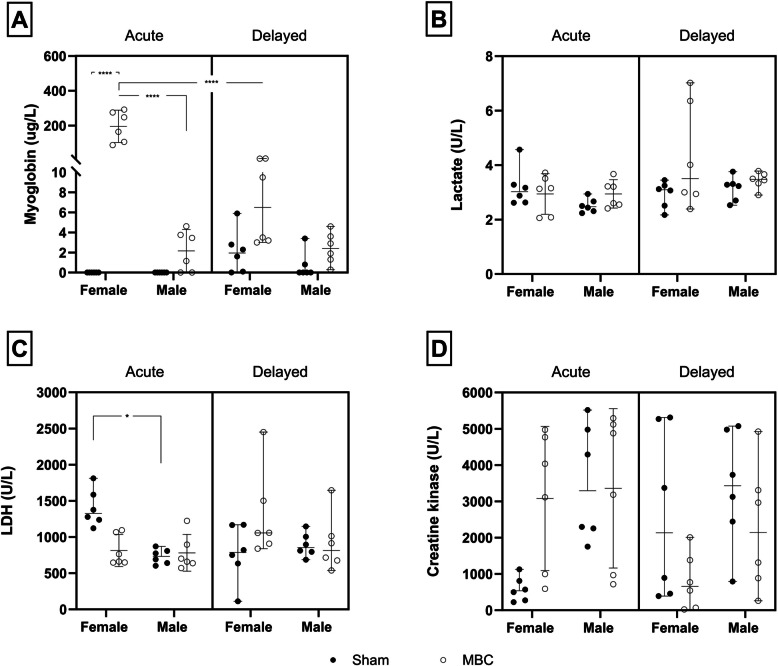
**A** Urine myoglobin (μg/L), **B** lactate (U/L), **C** LDH (U/L), and **D**) CK concentrations (U/L), in male and female rats of sham (black dots) and MBC-like-exposed rats (open circles); ****p < 0.0001; *p < 0.01. The error bars describe the 95% confidence interval

With respect to urine myoglobin concentrations (Fig. 4A), a significant three-way interaction between exposure, sex, and time was shown [*F*(1, 40) = 28.0, *p* < 0.0001]. Pairwise comparisons revealed female rats exposed to the MBC-like protocol to present with a significant acute increase in myoglobin concentration compared to their sham-exposed counterparts (0.0 ± 0.0 vs. 195.7 ± 88.1 µg/L, *p* < 0.0001, *d* = 3.1, *d*CI[1.3; 4.9]) and compared to male MBC-exposed rats (2.2 ± 2.0 µg/L, *p* < 0.0001, *d* = 3.1, *d*CI[1.3; 4.8]). Further, a significant reduction in these values were noted in female rats after the 14-day recovery period (6.8 ± 3.9 vs.195.7 ± 88.1 µg/L, *p* < 0.0001, *d* = 3.0, *d*CI[1.3; 4.7]).

For lactate (Fig. 4B), only time significantly impacted the result [*F*(1, 40) = 5.10, *p* = 0.03], although no significant pairwise differences between any of the groups or trends in a specific direction were shown.

For LDH (Fig. 4C), exposure, sex and time showed a significant interaction [*F*(1, 40) = 8.77, *p* = 0.005], with sham-exposed female rats presenting with significantly higher acute plasma LDH concentrations, compared to male sham-exposed rats (1401.7 ± 254.0 vs. 731.7 ± 103.6U/L, *p* = 0.034, *d* = 3.4, *d*CI[5.3; 1.5]). Also, MBC-like-exposed female rats presented with a noteworthy reduction in acute LDH concentrations (813.7 ± 211.4 U/L, *p* = 0.1, *d* = 2.5, *d*CI[0.9; 4.0]), compared to their sham-exposed counterparts. No differences were observed at the delayed timepoint.

While no three-way interaction was shown with respect to CK concentrations (Fig. 4D), a significant exposure-time interaction was shown [*F*(1, 40) = 7.32, *p* = 0.01]. Again, no significant pairwise differences were observed, although MBC-like-exposed female rats tended to present with higher acute CK concentrations compared to sham-exposed rats (3514.7 ± 1607.7 vs. 584.2 ± 340.3 U/L, *p* = 0.4, *d* = 1.8, *d*CI[0.4; 3.2]).

### Effect of MBC-like exposure on acute and delayed behavioural outcomes

**Fig. 5:**
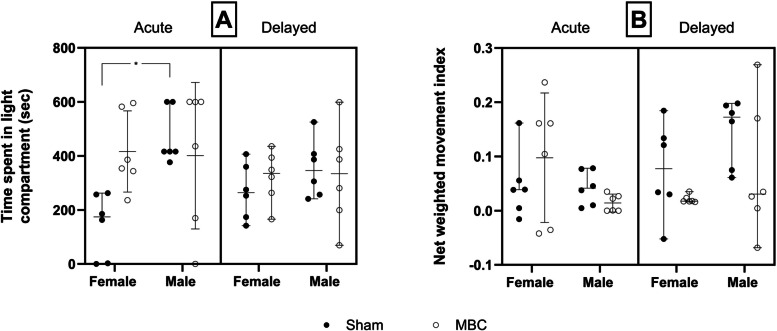
**A** Light–dark, and **B**) social behaviour, of male and female rats exposed to either sham (black dots) or MBC-like exposure (open circles); *p < 0.05. The error bars describe the 95% confidence interval

Neither the light-dark (Fig. 5A), nor social behaviour (Fig. 5B) of sham and MBC-like-exposed rats were impacted by significant three-way interactions. A significant exposure-sex interaction was shown with respect to the light-dark test [*F*(1, 40) = 6.01, *p* = 0.019]. Also, a significant time-sex interaction was shown for the social behaviour of rats [*F*(1, 40) = 5.98, *p* = 0.02]. Bonferroni post-hoc tests revealed that female sham-exposed rats spent significantly less time in the light compartment, compared to their sham-exposed male counterparts (145.1 ± 118.2 vs. 470.9 ± 101.1 s, *p* = 0.014, *d* = 2.7, *d*CI[1.2; 4.6]). Further, although missing statistical significance, MBC-like-exposed female rats trended towards spending more time in the light compartment immediately after MBC-like exposure, compared to shamexposed females (Fig. 5A; 416.3 ± 143.1 s, *d* = 1.9, *d*CI[0.6; 3.5]). No pairwise differences in social behaviour was observed in any of the groups compared at 15 days post-MBC-like exposure **(**Fig. 5B**)**.

### Effect of contextual trigger exposure on delayed urine myoglobin and skeletal muscle metabolites

**Fig. 6:**
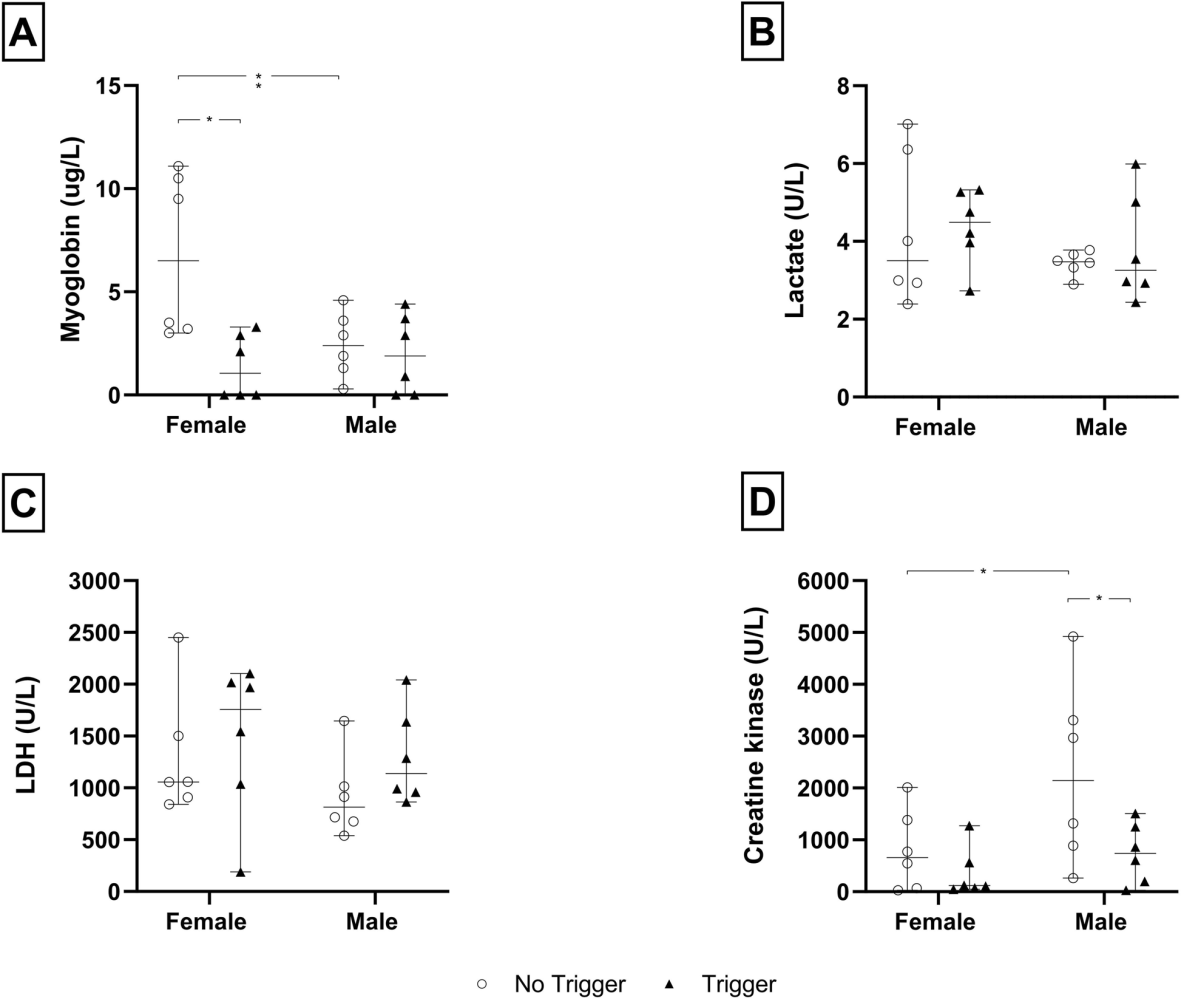
**A** Urine myoglobin (μg/L), **B** lactate (U/L), **C** LDH (U/L), and **D**) CK concentrations (U/L), in male and female rats under no-trigger (open circles) and trigger (black triangles) conditions; **p < 0.01; *p < 0.05. The error bars describe the 95% confidence interval

In these comparisons, data from MBC-like-exposed rats that were reassessed after the recovery period in the absence or presence of re-exposure to the aversive white noise (trigger condition), are shown. A significant condition-sex interaction was shown for urine myoglobin concentration only (Fig. 6A; *F*(1, 20) = 6.11, *p* = 0.022). Pairwise comparisons revealed female trigger-exposed rats to have significantly lower urine myoglobin concentrations compared to female rats in the no-trigger condition (1.4 ± 1.6 vs. 6.8 ± 3.9 µg/L, *p* = 0.0022, *d* = 1.8, *d*CI[0.4; 3.1]), while female no-trigger rats presented with significantly higher urine myoglobin concentrations compared to male rats in the no-trigger condition (2.4 ± 1.6 µg/L, *p* = 0.012, *d* = 1.5, *d*CI[0.1; 2.7]).

With respect to CK (Fig. 6D), both condition [*F*(1, 20) = 5.46, *p* = 0.03] and sex [*F*(1, 20) = 4.83, *p* = 0.04] significantly impacted the result, with male rats of the no-trigger condition having higher CK concentrations than female rats in the same-condition group (2277.0 ± 1759.3 vs. 800.7 ± 775.2 U/L, *p* = 0.045, *d* = 1.1, *d*CI[−0.2; 2.3]), and compared to male rats exposed to the trigger prior to testing (742.3 ± 581.5 U/L, *p* = 0.04, *d* = 1.17, *d*CI[−0.1; 2.4]). No statistically significant interactions were shown for lactate and LDH concentrations (Fig. 6B and C).

### Effect of contextual trigger exposure on delayed behavioural outcomes

**Fig. 7:**
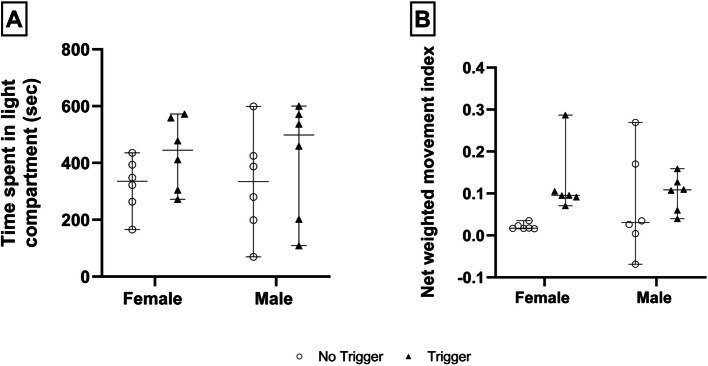
**A** light–dark, and **B**) social behaviour, of male and female rats under no-trigger (open circles) and trigger (black triangle) conditions. The error bars describe the 95% confidence interval

No significant two-way interactions, nor main effects of trigger-condition or sex were shown for either the delayed light–dark or social behaviour of rats (Fig. 7A and B). However, the trigger condition trended towards impacting the social behaviour of rats [*F*(1, 20) = 4.83, *p* = 0.05] (Fig. 7B), with trigger-exposed female rats tending to spend more time together, compared to female rats of the no-trigger condition (0.1 ± 0.1 vs. 0.02 ± 0.01, *p* = 0.622, *d* = 1.8, *d*CI[0.4; 3.2]).

## Discussion

Here we describe and characterise a novel rodent model system in which the ethological constructs of MBC-related pathophysiological and neurocognitive complications can be studied. Importantly 1) MBC-like exposure induced significant acute increases in rectal temperature in animals of both sexes and significantly increased urine myoglobin content in females compared to sham exposure, 2) female rats exposed to the MBC-like trended towards avoiding a dark environment more compared to sham-exposed rats, and 3) a contextual trigger resulted in significantly increased urine myoglobin and decreased plasma CK concentrations in females versus no-trigger MBC-like exposed rats. Moreover, males subjected to a contextual trigger showed a significant decrease in CK that was not observed in females.

Increased rectal temperature is a consistent physiological phenomenon seen in various species following severe stress, including mice [34, 35], rats [35, 36], antelope [5, 9], and humans [[8]. Here, both male and female SD rats presented with a significant increase in core body temperature compared to sham exposure (Fig. 3). Further, the average increases in core body temperatures reported (male: 2.4 °C; female 2.6 °C) are notably higher than the 0.8 – 1.5 °C values normally reported after moderate exercise [9, 37, 38], highlighting the effectiveness of the MBC-like protocol to act as a hyperthermic trigger and in line with our previous study [21]. Since myoglobinuria is a common manifestation of CM [5, 6], which is also commonly associated with a hyperthermic state [39], we show that MBC-like exposure results in rhabdomyolysis with a significant increase in urine myoglobin that is significantly more pronounced in female than male rats immediately after MBC-like-exposure **(**Fig. 4A). Thus, MBC induces greater disruption of muscle integrity in female rats which is of significant interest. We postulate that male rats can increase their metabolic energy turnover under conditions of MBC, thereby potentially recruiting a myoprotective mechanism [21], irrespective of robust changes in their body temperature.

CM is also characterised by increased plasma CK, LDH, and lactate concentrations [7, 40] that mostly stem from myocyte damage and increased cell membrane permeability [7, 40]. While we failed to highlight a significant main effect of MBC-like exposure on these parameters (Fig. 4B** – D)**, exposure did impact LDH and CK concentrations in concert with sex and time. This interplay is exemplified by the significant elevation in serum LDH in female, compared to male rats at baseline, but not after MBC-like exposure. Interestingly, LDH presents with at least five isoenzymes that are ubiquitously present in all tissues, most notably in cardiac and skeletal muscle [41]. Here, LDH is responsible for the reversible conversion of pyruvate to lactate under anaerobic conditions [42]. Since LDH is known to be elevated following high-intensity exercise [41], we cannot exclude the involvement of other LDH isoenzymes that could have yielded a false LDH status. Still, higher serum LDH concentrations at baseline commonly correlate positively with pathological severity in several conditions, including myopathology [41]. The rise in CK in sham group rats is interesting. But since this was not significant, we won’t speculate further on its origins. Nevertheless, our results point to muscle breakdown in female rats being associated with increased *baseline* LDH concentrations, and *post-exertion* CK concentrations. In contrast, serum LDH and CK concentrations remained unaltered in males as a function of MBC-like exposure, supporting the earlier conclusion that male rats are more resilient to the effects of forced exertion. That post-MBC reduced serum LDH in female rats, albeit non-significantly, the present results highlight sex dimorphism in the response of rats to forced exertion. Importantly, changes in serum biochemistry in females were mostly observed immediately after exertion, and not at 15 days post-MBC-like exposure. This emphasizes that LDH and CK may not be suitable markers for assessing long-term effects of forced exertion on myocyte function and that other biochemical avenues need to be considered. To elaborate further on how MBC-like exposure and the above biochemical changes may contribute to stress-related anxiety-like manifestations, we assessed avoidance behaviour using the LDT and SIT.

In the LDT, rats are known to show a natural preference for the dark compartment [26, 27, 43]. However, in the MBC protocol we introduced post-exertion social confinement as an additional stressor. This was done by enclosing three rats of the same sex in a small dark enclosure, which left little room to move, while retaining the aversive white noise in the background. Therefore, although paradoxical, aversion to the dark compartment could be expected under model conditions. MBC did not evoke any pronounced effects on anxiety in rats (Fig. 5A). Nevertheless, female rats trended towards avoiding a dark environment in the LDT immediately after exposure, compared to sham-exposed rats, and hence were more anxious (*d* = 2.7). This highlights a possible protracted anxiogenic effect of the MBC-like protocol that may have diminished over time post-acute stress. Further time-dependent studies are required to verify this assumption. In addition, too few animals per group (n = 6) may also have contributed. Our data are unclear in terms of whether male rats experienced heightened anxiety, especially since they showed more light-directed behaviour at baseline, although this failed to reach significance. The open field test, elevated plus or zero maze, may elucidate this result better. Nevertheless, the sex bias in these results largely agree with the biochemical findings, that female rats tend to be more affected by MBC than males.

The SIT is a generalised assessment of anxiety as a function of social interaction [29–31] with foreign conspecifics. It is used here for its resemblance of forced interaction with unknown conspecifics as it transpires in the wild. However, MBC-like exposure had no effect on the social behaviour of rats of either sex, with only time or sex interacting to influence the result (Fig. 5B). Future work will need to explore other forms of social performance in this model, especially breeding performance. Indeed, rats live and procreate mostly in closed communities [44], while reproduction in ungulates follows careful natural selection of mates. Studies of sociability in rats may therefore be an inappropriate post-MBC-marker of behavioural adaptation. This notion needs further study. Since there are no published pre-clinical animal models of MBC-related CM models in rodents, comparing and discussing our behavioural findings with similar work is not possible. Moreover, the chemically induced rhabdomyolysis models [18–20], which are somewhat ineffective to elaborate our understanding of CM, have not considered behavioural analyses, which is unfortunate.

In many ways the prognosis and later sequelae of CM in risk prone animals are typically experienced after trauma, where inappropriate consolidation of the traumatic context (sound, sight, smell, touch, taste) promotes conditioned fear responses post-trauma [45, 46]. To this end, so-called ‘contextual reminders’ play an important role as environmental triggers [47–49]. This phenomenon is also proposed to contribute to the delayed consequences of MBC [50], which we attempted to replicate by re-exposing rats to the original aversive sound 15 days later. Regarding the biochemistry data, the sex-specific variations observed immediately after the MBC-like procedure were not evident post-trigger exposure on day 15, irrespective of the parameter measured. That said, a significant reduction in urine myoglobin concentration was observed in trigger-exposed, compared to non-re-exposed female rats, although the overall concentrations of myoglobin in both groups were in the range of sham-exposed females immediately after the MBC-like procedure (i.e., 0 – 10 µg/L; Fig. 6A). Why female trigger-exposed rats have significantly lower urine myoglobin concentrations compared to non-trigger exposed females is intriguing. While speculative, an increase in myoglobin in the MBC plus no trigger (aversive noise) group may reflect a gradual upregulation of myoglobin transport to the muscles post MBC-induced exercise. Alternatively, a higher-than-normal oxygen demand and oxygen metabolism after MBC-induced exertion, especially since applied till the point of exhaustion, will increase reactive oxygen intermediates and so prompt an increase in myoglobin (a free radical scavenger; [51]) as an antioxidant defense mechanism. Regarding behaviour, the contextual trigger only affected social behaviour of female rats, which trended towards spending more time together after trigger exposure, compared to rats that were not contextually reminded (*d* = 1.8) **(**Fig. 7B**)**. However, since MBC-like stress alone did not impact social behaviour of female rats at the time of MBC-like exposure, it cannot be concluded with certainty that the sound trigger acted as a stressor rather than a contextual reminder to drive safety-seeking social interactive behaviour. Further studies are therefore needed to establish the role of preconditioning in the development of anxiety-like behaviour in MBC-rats later in life.

Taken as a whole, our data highlight sex-dimorphism in the post-MBC-like biochemical and behavioural changes in rats. This is noteworthy as we have earlier noted that female rats are more prone to develop specific signs of CM, with male rats seemingly being more resilient to the effects of MBC-like exposure [21]. Testosterone is well documented to promote muscle growth and strength, which has specific relevance to the pathology of CM [52]. Although it would remain difficult to translate findings from rodent studies to wildlife, the sex-dependent differences described here hint at differential influences of sex hormones on the biobehavioural impact of forced exertion that should be explored in future work. Studies looking at interactions between myocyte fibre composition and sex hormones within the context of adaption to forced exertion, would also provide valuable insights. This study builds on our previous research using a rodent model of CM, which confirmed that the running protocol effectively elicited the necessary muscular response. It emphasizes hyperthermia and increased urine myoglobin, particularly in females, highlighting sex-specific physiological responses. By incorporating behavioural and biochemical assessments, acute versus delayed response, and the role of environmental triggers, the study enhances model validity and deepens insights into CM-related responses.

Regarding limitations, while the measurement of various known biomarkers associated with CM (see [52]) added significantly to biological understanding of the model, protocols that consider more appropriate times for biomarker collection should be considered. This is especially important since we cannot exclude ‘blind spots’ in the presently applied windows of sampling, or that other biochemical adaptations did not transpire. Further, analysis of monoamines and corticosterone could have added to the validity of our model and provide an important link between CM and maladaptive stress responses, especially in female rats. Although the absence of a sex-based biological screen is noted, e.g., estrogen, the interaction between female sex, biological traits, and skeletal muscle function and integrity, would be more valuable. Finally, drug validation studies using known capture drugs would shed light on the validity and utility of this model.

## Conclusion

The present work is potentially informative of our understanding of CM, specifically with the protocol emphasizing forced muscular exertion. This study extends our earlier research by highlighting sex dimorphism and contextual factors in MBC-like exposure. Both male and female rats subjected to an MBC-like protocol present with significant post-exercise hyperthermia and increased urine myoglobin concentrations. However, sex dimorphism may be evident in the biological and behavioural sequelae of MBC-like exposure, with females more sensitive and males more resilient. Future work is needed to fully elucidate our understanding of CM in captured wildlife. To this end, this model might provide useful insights.

## Data Availability

The datasets analysed within the current study are available from the corresponding author upon request.
